# Thyroid dysfunction and cardiovascular disease

**DOI:** 10.1093/eurheartj/ehag248

**Published:** 2026-04-17

**Authors:** Heba Alwan, Ola Hysaj, Baris Gencer, Leonidas Duntas, Nicolas Rodondi

**Affiliations:** Institute of Primary Health Care (BIHAM), University of Bern, Bern 3012, Switzerland; Department of Endocrinology, Diabetes and Metabolism, Lausanne University Hospital, University of Lausanne, Lausanne 1011, Switzerland; Institute of Primary Health Care (BIHAM), University of Bern, Bern 3012, Switzerland; Graduate School for Health Sciences, University of Bern, Bern, Switzerland; Institute of Primary Health Care (BIHAM), University of Bern, Bern 3012, Switzerland; Department of Cardiology, Lausanne University Hospital (CHUV) and University of Lausanne, Lausanne 1011, Switzerland; Thyroid Section, Unit of Endocrinology, Diabetes and Metabolism, Evgenideion Hospital, National and Kapodistrian University of Athens, Athens, Greece; Institute of Primary Health Care (BIHAM), University of Bern, Bern 3012, Switzerland; Department of General Internal Medicine, Inselspital, Bern University Hospital, University of Bern, Bern 3010, Switzerland

**Keywords:** Thyroid dysfunction, Subclinical thyroid dysfunction, Cardiovascular disease, Levothyroxine, Cardiovascular risk

## Abstract

Thyroid hormones play an important role in regulating the cardiovascular system. Thyroid dysfunction, which encompasses hypothyroidism and hyperthyroidism, is relatively common and has been linked to a range of cardiovascular manifestations, including dyslipidaemia, abnormal blood pressure regulation, endothelial dysfunction, impaired cardiac function, and arrhythmias. The effects of levothyroxine replacement therapy on cardiovascular endpoints in subclinical hypothyroidism remain uncertain. The largest randomized controlled trial (TRUST) to date was underpowered to assess cardiovascular outcomes, although the results demonstrated a pattern towards a beneficial effect of levothyroxine. The aim of this review was to provide a summary of current knowledge on the relationship between thyroid dysfunction and cardiovascular health, with a focus on molecular and pathophysiological mechanisms underlying thyroid dysfunction and cardiovascular disease. Evidence from recent omics and Mendelian randomization studies is discussed, alongside an overview of the epidemiology of thyroid diseases and the impact of levothyroxine treatment on cardiovascular outcomes in subclinical hypothyroidism.

## Graphical Abstract

**Graphical Abstract:**
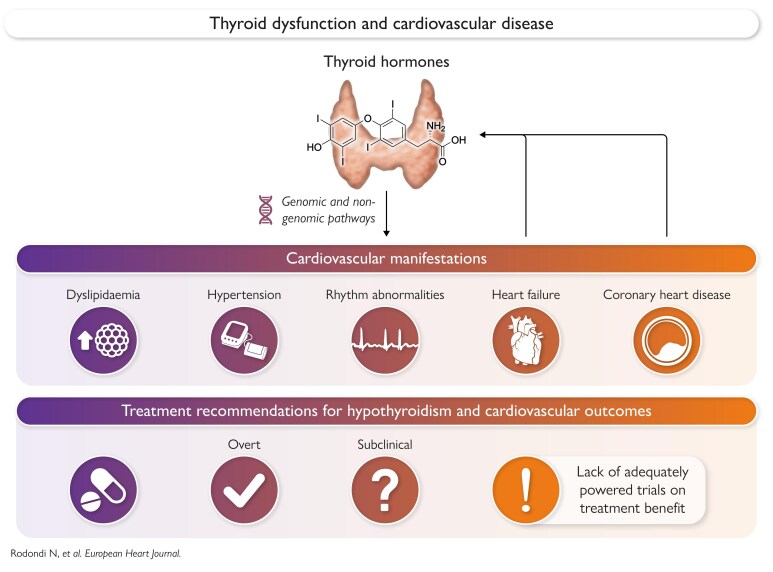

Cardiovascular manifestations of thyroid dysfunction.

Cardiovascular diseases (CVDs) remain the leading cause of mortality worldwide, with the overall burden projected to increase over the coming decades.^1^ The impact of thyroid disorders on cardiovascular health has long been discussed.^2^ The aim of this review was to provide a comprehensive and up-to-date summary of the relationship between thyroid dysfunction and cardiovascular health, spanning molecular mechanisms and pathophysiology through to epidemiological evidence. It extends previous reviews by incorporating recent data, including emerging findings from multi-omics and Mendelian randomization (MR) studies.

## Thyroid hormones and the cardiovascular system: molecular mechanisms

Thyroid status is determined by measuring thyroid function tests in peripheral blood. *Table 1* classifies thyroid status using the current reference ranges for serum thyroid-stimulating hormone (TSH), free thyroxine (FT4), and free triiodothyronine (FT3) concentrations (further details are provided in the Supplementary Materials**).**

**Table 1:** Commonly defined categories of thyroid status

Thyroid status	TSH^b^	Free T4	Free T3	Causes
**Overt Hyperthyroidism**	Low	Elevated	Elevated or normal	Exogenous cause: excessive thyroid hormone replacement. Endogenous Causes: Graves’ disease, multinodular goitre, an autonomously functioning thyroid nodule or Hashitoxicosis.
**T3 Toxicosis**	Low	Normal	Elevated	Graves’ disease, multinodular goitre, or an autonomously functioning thyroid nodule.
**Subclinical Hyperthyroidism**	Low	Normal	Normal	Exogenous cause: excessive intake of thyroid hormone or desiccated thyroid extract. Endogenous Causes: Graves’ disease, multinodular goitre, or an autonomously functioning thyroid nodule.
**Euthyroid (Normal)**	Normal	Normal	Normal	NA
**Sick Euthyroid Syndrome (Non-Thyroidal Illness Syndrome)^a^**	Normal or Low	Normal or Low	Low	Primarily triggered by acute or chronic systemic illnesses, including sepsis, cardiac disease and critical care settings.
**Overt Hypothyroidism**	Elevated	Low	Low or normal	Exogenous causes: Inadequate TH replacement or noncompliance for overt hypothyroidism. Endogenous causes: Hashimoto’s autoimmune thyroiditis, Thyroid damage due to thyroidectomy or radiation. Secondary hypothyroidism due to TSH deficiency.
**Subclinical Hypothyroidism**	Elevated	Normal	Normal	Exogenous causes: inadequate levothyroxine replacement therapy in patients with hypothyroidism, as well as the use of medications such as lithium, cytokines, iodine, antithyroid drugs, or interventions like radioiodine therapy and thyroidectomy. Endogenous causes: Hashimoto's thyroiditis, a history of subacute or silent thyroiditis and ageing (some elderly individuals may develop subclinical hypothyroidism due to age-related decline in thyroid function

TSH, thyroid-stimulating hormone; T4, thyroxine; T3, triiodothyronine; NA, not applicable.

^a^Non-thyroidal illness syndrome (NTIS) includes: Low T3 syndrome, Low free T3/T4 syndrome, or transient TSH suppression. NTIS reflects transient alterations in thyroid hormone levels during systemic illness and is not classified as not a primary thyroid dysfunction.

^b^ **Reference range variability:** TSH, FT4 and FT3 ranges vary by laboratory and assay method.

**Commonly used adult reference ranges** (non-pregnant): TSH: ∼0.4–4.4 mIU/L; Free T4: ∼10–23 pmol/L *(0.8–1.8 ng/dL)* Free FT3: ∼ 3.1–6.8 pmol/L *(2.0–4.4 pg/mL)* These values are provided for context only; interpretation in clinical practice should always rely on assay-specific reference intervals.

**Figure 1:**
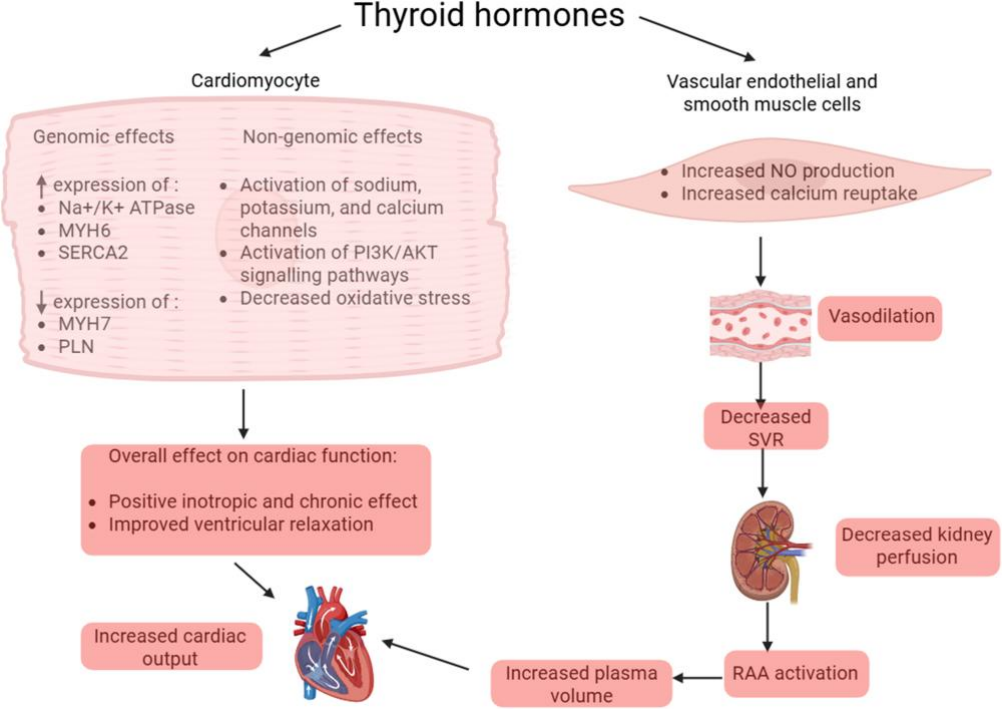
Molecular and physiological mechanisms underlying the effects of thyroid hormones on cardiomyocytes and vascular endothelial and smooth muscle cells, and their overall impact on the cardiovascular system. Created in BioRender. Alwan, H. (2026) https://BioRender.com/63528ga Na+/K1 ATPase: sodium-potassium adenosine triphosphatase; MYH6: myosin heavy chain α; SERCA2: sarcoplasmic/endoplasmic reticulum calcium ATPase 2; MYH7: myosin heavy chain β; PLN: phospholamban; PI3K/AKT: phosphatidylinositol 3 kinase/protein kinase B; NO: nitric oxide; SVR: systemic vascular resistance; RAA: renin-angiotensin-aldosterone axis

The role of thyroid hormones on cardiovascular physiology is particularly important with effects on both cardiomyocytes and vascular endothelium (*Figure 1*). The cardiac effects of thyroid hormones are primarily mediated via interaction with nuclear-based thyroid hormone receptors, which modulate the expression of certain cardiac proteins.^3–5^ The effects of thyroid hormones on the heart are primarily mediated by T3, which increases cardiac contractility, heart rate, and the speed of cardiac relaxation during diastole.^2,6^ Decreased ventricular relaxation, which leads to diastolic dysfunction as seen in persons with hypothyroidism, is believed to be secondary to the downregulation of the gene encoding sarcoplasmic/endoplasmic reticulum calcium ATPase 2 (SERCA2), and increased expression of phospholamban, which in turn causes a decrease in calcium uptake during diastole.^7,8^ Recent reports indicate that cardiomyocytes from hypothyroid female rats exhibit misalignment of cardiac transverse tubules, increased calcium sparks, and reduced co-localization of L-type calcium channels and junctophilin-2, which are essential for effective cardiomyocyte contractile activity.^9^ These alterations were improved following oral triiodo-L-thyronine administration.^9^ In contrast, excess thyroid hormone upregulates β-adrenergic receptor expression, leading to increased heart rate and cardiac output.^10^ This enhanced adrenergic responsiveness may subsequently contribute to the development or exacerbation of heart failure (HF) in the setting of hyperthyroidism.^10,11^ Experimental studies have demonstrated that both TSH and peripheral thyroid hormones exert regulatory effects on microRNA-1 and hyperpolarization-activated cyclic nucleotide-gated channel 2.^12,13^ These molecules have been implicated in the pathogenesis of cardiac hypertrophy, myocardial remodelling, and HF.^13–15^ In addition, thyroid hormones have been reported to modulate the expression and function of gap junction channels.^10,12^

Thyroid hormones also influence cardiomyocytes through non-genomic pathways primarily through modulation of ion channels and signalling pathways.^4,5^ In a rat model of central hypothyroidism, TSH directly mediated cardiac electrical remodelling by suppressing repolarizing potassium currents via TSH receptor-protein kinase A signalling. This effect increases arrhythmia risk through increased triggered activity.^16^

Moreover, thyroid hormones promote relaxation of vascular smooth-muscles cells leading to reduced systemic vascular resistance.^2,6^ Lower systemic vascular resistance reduces the effective arterial filling volume, which then increases renin release and activates the angiotensin-aldosterone axis.^2^ The latter results in increased renal sodium reabsorption and a rise in plasma volume. This process, in conjunction with the stimulation of erythropoietin secretion, contributes to the increase in blood volume and cardiac preload, thereby further augmenting cardiac output.^2^ In contrast, thyroid hormone deficiency leads to decreased production of nitric oxide, thereby increasing systemic vascular resistance.^7,17^

Research has shown that T3 stimulates the activity of 3-hydroxy-3-methylglutaryl coenzyme A reductase, which is essential for cholesterol production and a target of statin medications, while simultaneously upregulating hepatic low-density lipoprotein receptor gene expression.^10,18^ Moreover, thyroid hormones influence cholesterol 7α-hydroxylase, the enzyme responsible for cholesterol breakdown, and promote hepatic lipogenesis via activation of the carbohydrate response element-binding protein transcription factor.^10,19^ Reduced activity of lipoprotein lipase and lipid-related proteins is also observed in hypothyroidism.^10,20^

## OMICs

Omics research enables a comprehensive assessment of molecules at multiple levels, including the genome, transcriptome, proteome, and metabolome.^21,22^ Over the past decade, numerous omics studies have been carried out, providing important insights into the molecular links between thyroid dysfunction and CVD.^21^ A recent metabolomics study revealed that patients with hypothyroidism experienced significant reductions in circulating levels of ceramide, phosphatidylcholine, triglycerides, acylcarnitine, and peptides after achieving euthyroidism through levothyroxine (LT4) therapy.^23^ In a study involving 16 healthy men treated with LT4 for eight weeks to induce a hyperthyroid state, 65 metabolites and 63 proteins were associated with serum FT4 levels, and these markers correlated with elevated resting energy expenditure, enhanced protection against systemic oxidative stress, and reduced levels of lipoprotein particles.^24^ Supporting these observations, a population-based analysis from the Study of Health in Pomerania involving 952 participants identified significant associations between serum FT4 and 106 plasma metabolites, the majority of which were lipid-related, highlighting the possible effect of thyroid function on the lipid profile.^25^

Omics studies have therefore highlighted the effects of thyroid hormones on oxidative stress, lipid metabolism, and other related pathways. However, despite rapid progress in recent years, the clinical application of these findings remains limited. Very few of these biomarkers have been clinically approved, largely due to technical limitations in analysis, poor comparability across platforms, biological variability, and, in some cases, insufficient statistical power.^26^ In the future, translating these insights into practice could allow omics-based biomarkers to support early detection of CVD in patients with thyroid disorders, identify those who may benefit from treatment (e.g. for individuals with subclinical hypothyroidism), and guide personalized therapy, including responses to LT4 on lipid profiles and other cardiovascular risk markers.

## Pathophysiological mechanisms

### Thyroid dysfunction and dyslipidaemia

It is well established that overt thyroid dysfunction significantly influences lipid metabolism.^17^ Hyperthyroidism is typically associated with reduced serum cholesterol levels due to increased turnover, whereas hypothyroidism is linked to elevated cholesterol levels, particularly an increase in LDL-C concentrations due to reduced cholesterol clearance and breakdown of cholesterol.^17^ Hypothyroidism also increases oxidation of LDL-C and lipoprotein(a) levels, both of which are associated with atherogenesis.^10,17,27,28^ Additionally, reduced catabolism of triglycerides leads to increase serum triglyceride levels.^10,29^

### Thyroid dysfunction and cardiac rhythm abnormalities

In hypothyroidism, electrocardiographic (ECG) findings include sinus bradycardia, prolonged QTc interval, which may lead to Torsades de Pointes, ventricular tachycardia, low voltage, conduction abnormalities, T-wave inversion, and, rarely, atrioventricular block (*Table 2*).^4,6^ Rhythm abnormalities encountered in hyperthyroid states are sinus tachycardia, AF, short PR and QTc intervals, and ST segment elevation.^2,6^ Mechanisms linking hyperthyroidism to AF include increased left atrial pressure, ischaemia secondary to elevated resting heart rate, increased atrial ectopic activity, and shortening of the action potential duration.^30^

**Table 2:** Pathophysiological cardiovascular effects of thyroid dysfunction

System	Hypothyroidism	Hyperthyroidism
**Cardiac Rhythm Abnormalities**	Sinus bradycardia	Sinus tachycardia
	Prolonged QTc interval	Atrial fibrillation
	T-wave inversion	Short PR and QTc interval ST segment elevation
	Atrioventricular block	
**Dyslipidaemia**	Increased total cholesterol	Decreased total cholesterol
	Increased LDL-cholesterol	
	Increased lipoprotein(a)	Decreased LDL-cholesterol
	Increased triglycerides	
**Vascular Effects**	Diastolic hypertension	Systolic hypertension
	Increased carotid intima-media thickness	Elevated markers of endothelial dysfunction
**Heart Failure**	Diastolic dysfunction	Increased left ventricular mass
	Reduced cardiac output	Left ventricular dysfunction
	Pericardial effusion and non-pitting oedema	‘High-output’ heart failure
		Pulmonary hypertension

LDL, low-density lipoprotein.

These findings have been recently confirmed in a large registry-based study (*n* = 132 707) investigating the effect of thyroid dysfunction on ECG parameters.^31^ The study demonstrated that hyperthyroidism was associated with a higher heart rate as compared to euthyroid individuals.^31^ On the other hand, overt hypothyroidism was linked to a slower heart rate, increased P-wave duration, a longer PR interval, and low voltage.^31^ Furthermore, ECG changes associated with hyperthyroidism may potentially serve as an early diagnostic tool, as demonstrated in one study where researchers developed an artificial intelligence model capable of detecting hyperthyroidism using ECG data.^32^ The model accurately identified both subclinical and overt hyperthyroidism, and demonstrated that individuals identified by the algorithm had a significantly increased risk of all-cause mortality (hazard ratio (HR): 1.97, 95% confidence interval (CI): 1.73–2.24) and new-onset HF (HR: 2.21, 95% CI: 1.82–2.68).^33^

### Thyroid dysfunction and the vasculature

Although systolic blood pressure (BP) may be elevated in hyperthyroid states, the net effect on BP depends on the interplay between increased cardiac output and reduced systemic vascular resistance.^17^ Hyperthyroidism has also been found to be associated with elevated circulating markers of endothelial dysfunction.^34^ A 2020 study reported increased levels of vascular cell adhesion molecule-1 (1309 ± 292 vs 1009 ± 168 ng/mL, *P* < .001), which is associated with endothelial dysfunction, and lower ankle-brachial index (0.98 ± 0.11 vs 1.06 ± 0.10, *P* < .001) in patients with Grave’s disease, as compared to euthyroid individuals, although the clinical significance of these differences is likely modest.^35^ In patients with differentiated thyroid cancer undergoing thyroidectomy and radioiodine ablation, flow-mediated dilation (FMD) decreased significantly during the hypothyroidism state, reflecting endothelial dysfunction.^36^ Similarly, a study involving children with newly diagnosed Graves’ disease demonstrated elevated von Willebrand factor levels and impaired FMD of the brachial artery (*P* = .001).^37^ A study using computed tomography angiography found that individuals with overt hyperthyroidism had significantly higher mean coronary calcium scores (456.5 vs 199.5 vs 155.9; *P* < .0001) and more high-grade coronary artery stenoses (39.2% vs 24.2%; *P* = .007) as compared to euthyroid individuals.^38^

Overt hypothyroidism is associated with increased peripheral vascular resistance and diastolic hypertension.^17,39^ It has also been associated with increased carotid intima-media thickness and impaired endothelium-dependent vasodilation, largely due to reduced nitric oxide availability.^17,39^ A recent meta-analysis found an increase in pulse-wave velocity, reflecting increased arterial stiffness in persons with both overt and subclinical hypothyroidism.^40^ The arterial stiffness and endothelial dysfunction associated with both overt and subclinical hypothyroidism are thought to result from hyperlipidaemia and the pro-inflammatory state triggered by insufficient thyroid hormone levels.^17^ Additionally, the absence of T3-mediated vasodilatory effects contributes to increased arterial stiffness, which, in conjunction with a low-renin state, plays a role in the pathogenesis of hypertension in hypothyroidism.^17^ The suppression of the renin-angiotensin system in hypothyroidism was recently confirmed in a study conducted in Japan, where patients with thyroid carcinoma undergoing thyroid hormone withdrawal for radioactive iodine therapy exhibited significantly reduced plasma renin activity and elevated serum potassium levels four weeks after LT4 discontinuation.^41^

### Thyroid dysfunction and heart failure

Individuals with overt hyperthyroidism can present with symptoms of HF, such as exertional dyspnoea, even in the absence of prior cardiac disease.^2,4^ Overt hyperthyroidism enhances cardiac contractility, increases heart rate and cardiac preload, widens pulse pressure, and decreases systemic vascular resistance, all of which collectively contribute to an increased cardiac output.^17,42^ Severe, untreated hyperthyroidism can also lead to ‘high-output’ HF, resulting in pulmonary hypertension.^17^ Other proposed mechanisms contributing to HF in hyperthyroidism include increased left ventricular mass and left ventricular dysfunction, particularly in the context of AF.^10,43^ In older persons with pre-existing heart disease, the added strain caused by hyperthyroidism can further exacerbate cardiac dysfunction.^2^

In contrast, hypothyroidism has been associated with impaired ventricular filling and relaxation, which can lead to diastolic dysfunction.^10,17,44^ This condition also results in decreased cardiac output, primarily due to bradycardia, and can also lead to pericardial effusion. A meta-analysis of 11 case-control studies that examined changes in cardiac morphology and functional changes before and after supplementation with LT4 in individuals with subclinical hypothyroidism (*n* = 294) found that cardiac output and left ventricular ejection fraction were significantly increased after treatment.^45^

### Thyroid dysfunction and inflammation

Inflammation and oxidative stress are shared pathogenic features of thyroid disorders and CVD.^7,17^ A recent study demonstrated that patients with hyperthyroidism had higher levels of oxidative stress markers, which significantly decreased after treatment.^46^ Conversely, increased levels of levels of pro-inflammatory cytokines have been reported among patients with Hashimoto thyroiditis^47^ and treatment of hypothyroidism has been shown to reduce inflammatory markers.^48^

## Thyroid dysfunction and cardiovascular risk: epidemiologic evidence

**Figure 2:**
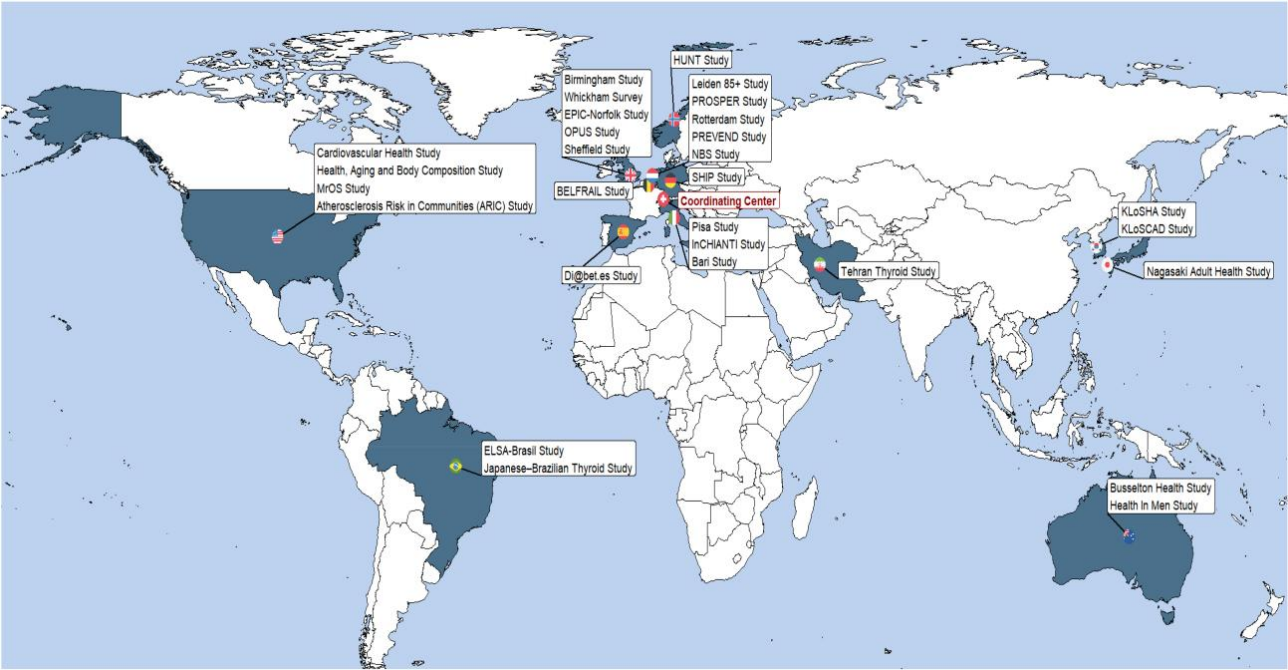
Thyroid Studies Collaboration map

Previous studies have extensively explored the effects of thyroid dysfunction on the cardiovascular system.^49–56^ Furthermore, a growing body of observational data suggests that cardiovascular risk may be increased even in individuals with subclinical thyroid dysfunction (SCTD). To address uncertainties in this area, the Thyroid Studies Collaboration (TSC) (https://www.thyroid-studies.org/; *Figure 2*), a large international consortium, was established.^56–58^ The TSC includes individual participant data (IPD) from 29 prospective cohorts, comprising over 167 000 participants across four continents, providing a unique opportunity to study adverse outcomes associated with SCTD.^53,55–58^ The following section provides a comprehensive synthesis of current evidence on the association between thyroid dysfunction and cardiovascular outcomes (summarized in *Table 3*).

**Table 3:** Evidence map of Epidemiologic Studies on Thyroid Dysfunction and Cardiovascular endpoints

Outcome	Thyroid Status	Study Type	Key Studies	Direction of association	Strength of evidence
**Atrial Fibrillation**	Overt hyperthyroidism	Observational, (meta-analysis and MR)	**Meta-analysis** of 5 studies (Huang *et al*., 2022): → (RR, 2.35; 95% CI, 1.07–5.16)—markedly increased AF risk **2 MR studies** reported hyperthyroidism is associated with a very small increased risk of AF OR, 1.05; 95% CI, 1.03–1.08 (Larson et al., 2019) OR, 1.05; 95% CI, 1.02–1.08 (Ruan et al., 2024)	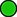 **Increased Risk**	Strong (consistent observational, observational data effect size: Large; MR effect size: very small (but precise and consistent))
	Subclinical hyperthyroidism	Observational, (IPD analysis, meta-analysis)	**IPD -analysis** of 5 cohorts (Collet *et al*., 2012): →TSH 0.10–0.44 mIU/L: HR 1.68 (95% CI: 1.16–2.43)—increased risk of AF. →TSH <0.10 mIU/L: HR 2.54 (95% CI: 1.08–5.99)—increased risk of AF. **Meta-analysis** of 13 cohorts (Huang *et al*., 2022): → RR 1.70 (95% CI: 1.11–2.62)—increased risk of AF.	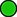 **Increased Risk**	**Strong** (consistent findings across IPD and aggregate-data meta-analysis, dose-response across TSH levels, large and clinically meaningful effect sizes)
	Subclinical hypothyroidism	Observational (IPD analysis, meta-analysis)	**Meta-analysis** of 7 cohorts (Singh *et al*., 2024): → RR 1.19 (95% CI: 1.03–1.39)—modest increased risk of AF **Meta-analysis** of 13 cohorts (Huang *et al*., 2022): → RR 1.23 (95% CI: 1.05–1.44)—modest increased risk of AF IPD analysis of 11 cohorts (Baumgartner et al., 2017): →TSH 7.0–9.9 mIU/L: HR 1.02 (95% CI: 0.73–1.41) →TSH 10.0–19.9 mIU/L: HR 0.94 (95% CI: 0.61–1.47)	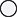 **Mixed evidence**	**Limited–Moderate** (modest effect sizes in meta-analyses, but inconsistency with IPD that has further adjusted for cardiovascular risk factors and excluded thyroid medication users)
	Overt hypothyroidism	Observational (cohort, meta-analysis)	**Meta-analysis** of 5 studies (Huang *et al*., 2022), no increased risk of AF: → RR 1.20 (95% CI: 0.72–1.99) Meta-analysis of 4 studies (Ning et al., 2017), no increased risk of AF: → RR 1.02 (95% CI: 0.71–1.46)	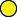 **No evidence of increased risk**	**Limited–Moderate** (consistent null findings across multiple meta-analyses of observational studies but limited by imprecision with broad CI).
**CHD**	Overt hyperthyroidism	Observational (meta-analyses)	**Meta-analysis of** 7 studies *(*Brandt *et al*., 2011), increased risk of CHD: → CVD mortality: RR 1.21 (95% CI: 1.05–1.38) **Meta-analysis** of 4 cohorts (Sohn *et al*., 2020), increased CHD risk: → CHD events: HR 1.11 (95% CI: 1.03–1.19) → CHD mortality: HR 1.24 (95% CI: 1.07–1.45)	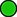 **Increased Risk**	**Moderate** (consistent observational data, modest or small effect sizes, precise estimates)
	Subclinical hyperthyroidism	Observational (IPD analysis, meta-analysis)	**IPD analysis** of 10 cohorts (Collet *et al*.,2012), increased risks of: → CHD mortality: HR, 1.29 (95% CI: 1.02–1.62) → CHD events. HR, 1.21 (95% CI: 0.99–1.46). **Meta-analysis** (Sohn *et al*., 2020), increased risk of → CHD events (11 studies): HR, 1.26 (95% CI, 1.06–1.49) → CV mortality (14 studies): HR, 1.21 (95% CI, 0.88 −1.67).	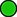 **Increased Risk**	**Moderate** (consistent observational data, small to moderate effect sizes, dose-response across TSH levels)
	Subclinical hypothyroidism	Observational (IPD analysis, meta-analysis)	**IPD analysis** of 11 cohorts (Rodondi *et al*., 2010); subclinical hypothyroidism is associated with increased risk of CHD: → (TSH ≥10): CHD events HR 1.89 (95% CI: 1.28–2.80) → (TSH ≥7): CHD mortality HR 1.42 (95% CI: 1.03–1.95) **Meta-analysis** of 16 studies (Sun *et al*., 2017) →CHD events: RR 1.27 (95% CI: 0.91–1.58)—increased CHD risk →CHD mortality: RR 1.37 (95% CI: 1.03–1.84) −increased CHD mortality risk	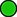 **Increased Risk**	**Moderate–Strong** (consistent observational data; dose-response across TSH levels)
	Overt hypothyroidism	Observational, (meta-analysis) and Genetic (MR)	**Meta-analysis** (Ning *et al*., 2017): → CVD mortality (17 studies): RR 1.96 (95% CI: 1.33–2.80)—markedly increased CVD mortality risk → CHD (13 studies): RR 1.13 (95% CI: 1.01–1.26)—modest increased CHD risk **MR evidence:** → MI: OR 1.02 (95% CI: 1.01–1.04)—very small causal effect (Li et al., 2024) → No evidence of a causal association with CHD (Ruan et al., 2024; Larsson et al., 2019)	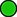 **Increased Risk**	**Limited–Moderate** (strong observational association for CVD mortality with a modest but consistent association for CHD incidence, inconsistent causal inference findings with very small effect size)
**Stroke**	Overt hyperthyroidism	Observational (meta-analyses, cohort)	Meta-analysis of 11 studies (Sohn *et al*., 2020): → HR 1.35 (95% CI: 1.03–1.75)—increased risk of stroke **Cohort study** (Sheu *et al*., 2010): → HR 1.44 (95% CI: 1.02–2.12)—increased risk of ischaemic stroke **Cohort study** (Kim *et al*., 2020). → Ischaemic stroke: HR 1.18 (95% CI 1.04–1.26)—increased risk of stroke → Hemorrhagic stroke: HR 1.13 (95% CI 0.88–1.47)- no evidence of increased risk	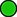 **Increased Risk**	**Moderate** (consistent association of increased risk, small to moderate effect sizes)
	Subclinical hyperthyroidism	Observational, (meta-analysis)	**Meta-analysis of 4 studies** (Chaker *et al*., 2014): → HR 1.17 (95% CI: 0.54–2.56)—no evidence of increased risk of stroke **Meta-analysis of 5 studies** (Sohn *et al*., 2020): → HR 1.17 (95% CI: 0.90–1.52)—no evidence of increased risk of stroke	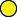 **No evidence of increased risk**	**Limited–Moderate** (consistent null findings, limited by small number of studies and imprecision with wide CIs, no subtype of stroke analysis)
	Subclinical hypothyroidism	Observational, (IPD analysis, meta-analysis)	**IPD analysis of 17 cohorts** (Chaker *et al*., 2015): → stroke events: HR 1.05 (95% CI: 0.91–1.21)—no evidence of increased risk →stroke mortality: HR 1.07 (95% CI, 0.80–1.42)—no evidence of increased risk → Age 18–49 years: Stroke events HR 3.32 (95% CI: 1.25–8.80) → Stroke mortality increased in younger age groups: Age 18–49 years: HR 4.22 (95% CI: 1.08–16.55) Age 50–64 years: HR 2.86 (95% CI: 1.31–6.26); (*P* for trend = 0.04) **Meta-analysis of 4 studies** (Chaker *et al*., 2014): → HR 1.08 (95% CI: 0.87–1.34)—no evidence of increased risk of stroke	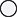 **Mixed evidence**	**Limited–Moderate** (consistent null findings in the overall population with small effect sizes and precise estimates for overall, large effect sizes for subgroup analysis of age, absence of analysis by stroke subtype)
	Overt hypothyroidism	Observational, (meta-analysis) and Genetic (MR)	**Meta-analysis** of 9 studies (Ning *et al*., 2017): → RR 1.09 (95% CI: 0.96–1.24) –no evidence of increased risk of stroke **MR study (**Tian *et al*., 2024): → Ischaemic stroke: OR 2.24 (95% CI: 1.18–4.26)—evidence supporting a causal association with overt hypothyroidism	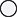 **Mixed evidence**	**Low to moderate** (inconsistent results across studies, null observational findings. MR study suggests a substantial causal effect for overt hypothyroidism, broad CI for MR)
**Heart Failure (HF)**	Overt hyperthyroidism	Observational, (meta-analysis)	**Meta-analysis of 4 studies** (Sohn *et al*., 2020): → HF risk: HR 1.28 (95% CI: 1.15–1.43)—increased HF risk **Meta-analysis** (Fan *et al*., 2024; *n* = 30 889): → HF occurrence among patients with overt hyperthyroidism: 8% developed heart failure	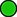 **Increased Risk**	**Moderate** (consistent results across studies, moderate effect sizes but did not account for thyroid treatment, precise estimates)
	Subclinical hyperthyroidism	Observational, (IPD analysis, meta-analysis)	**IPD analysis** of 6 cohorts (Gencer *et al*., 2012): → TSH <0.10 mIU/L: HR 1.94 (95% CI: 1.01–3.72)—increased HF risk, Evidence of a dose–response relationship with decreasing TSH levels **Meta-analysis** of 4 studies (Sohn *et al*., 2020): → HR 1.41 (95% CI: 0.80–3.30)—no statistically significant association	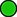 **Increased Risk**	**Moderate** (consistent direction of increased risk, effect sizes moderate to large, wide CIs, dose-response across TSH levels)
	Subclinical hypothyroidism	Observational, (IPD analysis, cohort, meta-analysis)	**IPD analysis** of 6 cohorts (Gencer *et al*., 2012): → TSH ≥10 mIU/L: HR 1.86 (95% CI: 1.27–2.72)—increased risk of HF events **Cohort study** (Rodondi *et al*., 2005): → TSH ≥7.0 mIU/L: more than 2-fold increased risk of HF events **Meta-analysis of 14 studies** (Yang *et al*., 2019); thyroid dysfunction and prognosis in heart failure: → All-cause mortality: HR 1.45 (95% CI: 1.26–1.67)– increased mortality risk → Cardiac death and/or HF hospitalization: HR 1.33 (95% CI: 1.17–1.50)—increased risk	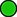 **Increased Risk**	**Moderate–Strong** (High-quality IPD analysis, consistent findings with cohort, large effect sizes, clear dose-response across TSH levels)
	Overt hypothyroidism	Observational, (meta-analysis, registry cohort)	**Danish national HF cohort (Petersen *et al*., 2024; *n* = 58 067):** → Composite outcome (mortality + rehospitalization): HR 1.24 (95% CI: 1.02–1.51) –increased risk among patients with overt hypothyroidism prior to HF onset **Meta-analysis of 8 studies (Ning *et al*., 2017):** → RR 1.13 (95% CI: 0.98–1.30)—no evidence of increased risk of HF	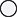 **Mixed evidence**	**Limited** (inconsistency between prognosis studies and heart failure risk and heterogeneity in exposure definitions, as thyroid hormone treatment was not accounted for in the meta-analysis but was excluded in the cohort study.)

AF, atrial fibrillation; HF, heart failure; IPD, individual participant data; CVD, cardiovascular disease; CHD, coronary heart disease; HR, Hazard Ratio; CI, confidence interval; RR, Risk Relative; Shyper, subclinical hyperthyroidism.

Shypo: subclinical hypothyroidism; TSH: thyroid-stimulating hormone, MR: mendelian randomization.

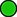
 = evidence of increased risk (consistent substantial and statistically significant increased risk across studies), 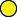 = no evidence of increased risk (no statistically significant relationship in most studies), 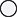 = mixed evidence (some evidence of increased risk, but inconsistent across studies).

**Strength of evidence** was evaluated by the authors using a narrative, non-formal approach based on consistency, study design and size, precision, control of bias and confounding, and supporting features such as dose–response patterns, and causal inference evidence (MR studies) rather than effect size alone.

### Dyslipidaemia

Overt hyperthyroidism is associated with lower total and LDL-cholesterol levels compared to euthyroid individuals.^32,59–61^ This association is supported by intervention studies reporting that treatment of overt hyperthyroidism is associated with worsening of the lipid profile. A meta-analysis of randomized and observational studies reported that treatment of overt hyperthyroidism leads to increases in lipid levels: total cholesterol increased by 44.5 mg/dL (95% CI: 38–51), and LDL-C by 31.1 mg/dL (95% CI: 24–38).^62^ In contrast, overt hypothyroidism is recognized as a major contributor to secondary dyslipidaemia with up to 90% of patients with hypothyroidism reported to have dyslipidemia.^2,7,29^ Elevated TSH levels are observed in 12%–13% of patients with hypercholesterolemia, significantly higher than the 2% prevalence observed in the general population.^63^ Furthermore, overt hypothyroidism is associated with an increase in lipoprotein(a) levels, a more potent marker of atherogenesis.^64^ Both observational studies and randomized controlled trials (RCT)s have demonstrated that treatment of overt hypothyroidism with LT4 can partially or completely normalize the lipid profile.^62^

The effect of subclinical hypothyroidism on lipid metabolism, however, remains more controversial.^65,66^ A large-scale study (over 11 000 participants) reported no significant differences in lipid parameters between individuals with subclinical hypothyroidism and euthyroid controls after matching for age and sex.^67^ Similarly, in a recent IPD analysis from the TSC (69 006 participants), there were no clear differences in lipid levels between individuals with SCTD and euthyroid individuals, with the exception of women with subclinical hyperthyroidism who had a lower LDL-C compared to euthyroid individuals (MD −0.17 mmol/L, 95%CI: −.29 to −.05).^65^ In contrast, a 2021 meta-analysis found that individuals with mild subclinical hypothyroidism (TSH < 10 mIU/L) had significantly higher levels of total cholesterol, LDL-C, and triglycerides, along with significantly lower high-density lipoprotein (HDL). These differences, however, are too small to explain the increased cardiovascular risk in subclinical hypothyroidism observed in previous studies.

Interventional studies of LT4 therapy for subclinical hypothyroidism have reported mixed results on lipid profiles.^68^ A 2020 meta-analysis demonstrated significant reductions in total cholesterol, LDL-C, and triglyceride levels after treatment in patients with both overt and subclinical hypothyroidism, with more pronounced improvements observed in those with overt hypothyroidism (decrease in total cholesterol by −58.4 mg/dL (95% CI: −64.70, −52.09) and LDL-C by −41.11 mg/dL (95% CI: −46.53, −35.69).^62^ Overall, the clinical significance of subclinical hypothyroidism treatment on lipids remains uncertain.

### Hypertension

Overt hyperthyroidism is identified as a secondary cause of hypertension and is associated with increased systolic BP, especially among individuals aged 50 years and older.^69,70^ The prevalence of hypertension in patients with thyrotoxicosis is high, estimated at 20–30%.^69^ Similarly, hypertension is common in overt hypothyroidism, with diastolic hypertension present in approximately 20% of hypothyroid patients.^71^

In contrast, the relationship between SCTD and hypertension remains unclear.^72,73^ A meta-analysis of cross-sectional studies found that subclinical hypothyroidism was associated with increased systolic and diastolic BP, whereas no such association was observed for subclinical hyperthyroidism.^72^ Results from a 2025 IPD analysis by the TSC did not show consistent differences in systolic or diastolic BP between individuals with SCTD and euthyroid individuals, with the exception of women with subclinical hyperthyroidism who had lower diastolic BP (MD: −1.3 mmHg, 95% CI: −2.0 to −.5) and men with subclinical hyperthyroidism who had lower systolic BP (MD: −3.1 mmHg, 95% CI: −4.8 to −1.4).^65^

A 2015 evidence review by the U.S. Preventive Services Task Force, based on two small randomized trials, found no significant BP–lowering effect of LT4 treatment.^68^ In contrast, a 2023 meta-analysis of 28 prospective cohort studies showed that LT4 treatment in individuals with subclinical hypothyroidism was associated with lower systolic BP (MD: −4.02 mmHg; 95% CI: −6.45 to −4.58) and diastolic BP (MD: −2.13 mmHg; 95% CI: −3.69 to −.56).^74^

### Atrial fibrillation

AF has been consistently associated with both overt and subclinical hyperthyroidism. Among patients with hyperthyroidism, the prevalence of AF ranges from 10% to 14%, significantly higher than the approximately 0.5% observed in the general population.^75^ A 2022 meta-analysis reported a marked increased risk of AF (relative risk (RR): 2.35; 95% CI: 1.07–5.16) among individuals with overt hyperthyroidism.^76^ Of note, treatment of hyperthyroidism does not guarantee restoration of sinus rhythm, with approximately one-third of patients continuing to have persistent atrial fibrillation.^77^ In a TSC IPD analysis, subclinical hyperthyroidism was associated with a 2.54-fold increased AF risk for TSH levels <0.1 mIU/L.^55^ Similar findings were observed in the Framingham Heart Study (for TSH *<* 0.1 mIU/L) and the Cardiovascular Health Study (for TSH *<*0.45 mIU/L), where older adults (≥60 years) with suppressed TSH had approximately 2–3 times higher AF risk. Increased AF risk remained significant even after adjusting for traditional cardiovascular risk factors such as systolic BP, smoking status, total cholesterol, and diabetes. Interestingly, increased risk of AF has been reported for high-normal FT4 levels even within the euthyroid range.^57,78^ On the other hand, the evidence on the association between subclinical hypothyroidism and AF is conflicting. Two meta-analyses reported increased risk, while an IPD analysis, after adjusting for cardiovascular risk factors and excluding thyroid medication users, found no evidence of increased risk.^57,76,79^ In contrast, overt hypothyroidism has not been associated with increased AF risk.^76,80^

### Coronary heart disease and cardiovascular mortality

Both overt and subclinical hypothyroidism have been associated with an increased risk of cardiovascular morbidity and mortality. Overt hyperthyroidism has been associated with a 16% increased risk of cardiovascular events and a 21% increase in cardiovascular mortality.^81,82^ A 2020 meta-analysis further confirmed increased CHD risk (HR 1.11; 95% CI 1.03–1.19) and cardiovascular mortality (HR 1.24; 95% CI 1.07–1.45) in overt hyperthyroidism.^83^ Similarly, a 2017 meta-analysis of 55 cohort studies reported a 22% higher cardiovascular mortality risk in overt hypothyroidism (RR 1.22; 95% CI 1.01–1.26).^80^

Insights into the effects of SCTD on cardiovascular outcomes are largely derived from two large IPD analyses conducted by the TSC. Subclinical hypothyroidism with TSH >10 mIU/L was associated with substantially increased CHD events (HR 1.89) and CHD mortality (HR 1.58).^56^ Subclinical hyperthyroidism was also associated with moderately increased risks of CHD events (HR 1.21) and CHD mortality (HR 1.29).^55^ These findings align with other meta-analyses reporting elevated CHD risk in both subclinical hypo- and hyperthyroidism.^8^ No increase in risk has been reported for TSH levels between 4.6–6.9 mIU/L.^72^ In addition to the degree of TSH elevation, age may also modulate cardiovascular risk, particularly in subclinical hypothyroidism. Longitudinal data show that TSH levels increase with age, and several observational studies suggest that subclinical hypothyroidism is associated with increased cardiovascular risk in younger, but not older, adults.^84–86^ However, TSC IPD analyses have not identified age as an effect modifier.^56^ Whether cardiovascular risk in subclinical hypothyroidism truly differs by age therefore remains a matter of debate.

### Heart failure

There is evidence supporting the association between thyroid dysfunction and increased risk of HF. In a 2020 meta-analysis, overt hyperthyroidism was associated with an increased risk of HF (HR: 1.28; 95% CI, 1.15–1.43).^83^ Similarly, a meta-analysis of 30 889 individuals with overt hyperthyroidism found that 8% developed HF.^87^ An IPD analysis from the TSC demonstrated a U-shaped relationship between TSH levels and HF risk. Participants with TSH concentrations ≥10 mIU/L and those with TSH <0.1 mIU/L had significantly increased risks of HF, with HR of 1.86 and 1.94, respectively, compared to euthyroid individuals.^58^ A recently published Danish registry-based study further highlighted the cardiovascular impact of thyroid dysfunction, reporting that both untreated overt and subclinical hypo- and hyperthyroidism were associated with an increased risk of mortality and hospitalization for HF among patients with newly diagnosed HF.^88^

### Stroke

Conflicting results have been reported on the association between thyroid dysfunction and stroke. Overt hyperthyroidism has been consistently associated with an increased risk of ischaemic stroke. Large cohort studies have reported that hyperthyroid adults have a moderately increased risk (up to 44%) of ischaemic stroke compared to euthyroid individuals, while no association was observed with haemorrhagic stroke (HR 1.13; 95% CI .88–1.47).^83,89^ These findings are supported by a meta-analysis of 11 cohort studies demonstrating a 35% increased risk of stroke in individuals with overt hyperthyroidism (HR 1.35; 95% CI 1.03–1.75).^83^ This association is biologically plausible given the prothrombotic state, increased atrial fibrillation burden, and adverse haemodynamic effects of excess thyroid hormone, which predominantly predispose to ischaemic rather than haemorrhagic stroke.^90,91^ In contrast, meta-analyses found no evidence of increased risk of stroke in subclinical hyperthyroidism.^83,92^ One IPD analysis using data from the TSC found no association between subclinical hypothyroidism and either stroke incidence or stroke-related mortality.^93^ However, age-stratified analyses suggested a potential age-dependent effect, with younger individuals (<50) showing a higher risk of stroke (HR 3.32 (95% CI: 1.25–8.80) whereas no association was observed in older adults.^93^ Overt hypothyroidism has not been associated with increased stroke risk, although available data remains limited.^80^

### Limitations of traditional observational studies

The variability observed across epidemiologic studies likely reflects substantial heterogeneity in population characteristics (e.g. age distribution and iodine status), differences in thyroid function assays and reference ranges, and inconsistent definitions of SCTD and cardiovascular endpoints. Furthermore, observational studies of overt thyroid dysfunction may have underestimated risk estimates, as overt thyroid dysfunction is more likely to be diagnosed and treated. As summarized in **Supplementary data online, *Table S1***, these sources of heterogeneity can meaningfully influence the magnitude and direction of associations with cardiovascular outcomes. Although IPD analyses improve analytical rigour by enabling standardized exposure and outcome definitions, harmonized covariate adjustment, and improved statistical power, their observational design inherently limits causal inference, and the resulting evidence is considered low- to moderate-quality evidence.

## Thyroid dysfunction and cardiovascular risk: evidence from Mendelian randomization studies

Observational studies are susceptible to confounding and reverse causation. While known confounders can be adjusted for, unmeasured factors may lead to spurious associations. Reverse causation can be illustrated with heart failure, where hypoxia-induced inflammation reduces cardiomyocyte deiodinase activity, lowering myocardial T3 levels.^7^ Moreover, a transient decline in serum thyroid hormones has been observed after acute MI and cardiac surgery.^7^

In recent years, MR studies have increasingly been used to test for causality, as they are less likely to be affected by these two issues as compared to conventional observational studies. MR uses genetic variants as instrumental variables to test whether an exposure is causally associated with an outcome.^94,95^ The introduction of gene arrays has facilitated large-scale genome-wide association studies (GWAS), which supply the summary data necessary for conducting MR analyses.^21^ The ThyroidOmics Consortium (www.thyroidomics.com) integrates genomic and multi-omics data from 34 independent cohorts and has enabled extensive meta-analyses of GWAS focused on thyroid function traits.^96^

It has been shown that genetically predicted lower TSH levels and hyperthyroidism are associated with an increased risk of atrial fibrillation with some studies suggesting height as a potential mediating factor.^97,98^ Conversely, the association between thyroid function and other cardiovascular outcomes in MR analyses has been less consistent. Findings from a bidirectional two-sample MR study published in 2024 identified hypothyroidism as a potential risk factor for coronary artery disease, angina pectoris, myocardial infarction (MI), and small vessel ischaemic stroke but did not find evidence of a causal association from atherosclerotic CVD to hypothyroidism.^99^ Consistently, another MR study reported a significant association between genetically predicted hypothyroidism and an increased risk of ischaemic stroke.^96^ A study published in 2020 reported an association between increasing levels of TSH within the normal range and reduced risk of stroke, with atrial fibrillation acting as a possible mediating factor.^100^ However, several other MR studies did not observe any causal relationships between thyroid function and other cardiovascular outcomes.^97,98^ In the opposite direction, a recent reverse MR study identified five plasma metabolites and sixteen immune cell traits that are causally associated with hypothyroidism risk.^98^

However, MR studies are not without limitations and the absence of an observed association between thyroid function and certain cardiovascular outcomes in MR analyses does not necessarily imply that no causal relationship exists. Moreover, the validity of MR analyses depends on several key assumptions. Inappropriate selection of genetic variants as instruments, the presence of horizontal pleiotropy (when genetic variants influence the outcome through pathways independent of the exposure), or linkage disequilibrium (non-random association of alleles at different loci) can introduce bias into the results.^99,101^ Finally, MR analyses may sometimes lack statistical power for certain outcomes, and it is important to interpret MR findings alongside evidence from other study designs, as MR alone cannot establish causality.^101^

Nevertheless, genetic insights gained from MR studies can help guide precision medicine, enabling personalized strategies for prevention and treatment of CVD while accounting for an individual’s thyroid function.^102^ Thyroid function tests exhibit low intraindividual variability but significant interindividual variation, which may reflect differences in each person's hypothalamic-pituitary-thyroid axis set point.^10,103^ However, the extent to which variation in this set point influences an individual’s cardiovascular risk remains unclear and warrants further investigation.^10^

## Effects of hormone replacement therapy in CVD

Substantial evidence supports the beneficial effects of LT4 treatment on cardiovascular risk in overt hypothyroidism.^104^ Several cardiac manifestations of overt hypothyroidism can be reversed by LT4 treatment, including hyperlipidaemia, hypertension, increased carotid intima-media thickness, decreased synthesis of endothelial-derived nitric oxide, and HF.^2,10^ It is important to acknowledge that conducting interventional studies in overt hypothyroidism is challenging due to ethical considerations, as current clinical guidelines universally recommend LT4 treatment for this group of patients.^45,105^

Conversely, robust evidence regarding the effect of treatment of subclinical hypothyroidism on cardiovascular outcomes remains limited, primarily due to the predominance of data derived from observational studies and small-scale interventional trials.^31^ When considering LT4 therapy, it is essential to carefully weigh the potential benefits against the risks of overtreatment, particularly in older adults.^7,106^ Adverse health outcomes associated with overtreatment include iatrogenic thyrotoxicosis, which can lead to atrial fibrillation notably in older adults, and accelerated bone loss in particular among postmenopausal women.^107^ A recent meta-analysis reported that approximately 20% of patients receiving LT4 therapy were overtreated.^108^ These findings underscore the importance of adequately monitoring thyroid function tests among individuals undergoing LT4 treatment. Moreover, management of thyroid disorders in the context of pre-existing CVD warrants particular attention. Special care is required when treating subclinical hypothyroidism in patients with heart failure, as overtreatment may precipitate adverse events.^7^ Conversely, the potential clinical benefit of T3 therapy after cardiac surgery or acute MI remains uncertain.^7,10^

In 2017, the largest RCT to date (TRUST), was published, assessing the effects of LT4 therapy in 737 older adults with subclinical hypothyroidism.^109^ The IEMO trial (*n* = 105) replicated the TRUST protocol in individuals aged 80 years and older.^110^ Both studies were underpowered to detect differences in cardiovascular events and mortality.^109,110^ Nevertheless, the two trials demonstrated a pattern towards a potential beneficial effect of LT4 on cardiovascular events with an HR of 0.89 (95% CI: .47–1.69) in the TRUST trial and an HR of 0.61 (95% CI: .24 to 1.50) in the IEMO trial.^109,110^ A 2024 IPD analysis of data primarily derived from the TRUST and IEMO trials found no significant difference in cardiovascular outcomes in individuals over 65 years old, regardless of LT4 therapy (HR 0.89, 95% CI .71–1.12).^111^

A recently published population-based cohort study utilizing data from the United Kingdom Clinical Practice Research Datalink reported that LT4 treatment in individuals with subclinical hypothyroidism was associated with a reduced risk of major adverse cardiovascular events (HR 0.88; 95% confidence interval 0.83–0.93).^112^ This analysis included 76 946 LT4-treated patients matched to an equal number of untreated individuals. A systematic review investigating the effects of LT4 on cardiovascular outcomes in individuals with coexisting subclinical hypothyroidism and HF identified only two eligible studies, consisting of an RCT and a nationwide cohort study, and was therefore unable to draw reliable conclusions, further underscoring the paucity of high-quality evidence in this area.^113^ Similarly, a TRUST trial analysis examining the effects of LT4 on systolic and diastolic heart function among 185 older adults with subclinical hypothyroidism found no difference after treatment with LT4 compared with placebo.^114^ Another approach to address the evidence gaps regarding the effect of LT4 treatment on cardiovascular outcomes, beyond meta-analyses, would be to apply emulation methods using data from both RCTs and cohort studies.

Evidence on the effects of LT4 treatment on surrogate markers of cardiovascular outcomes in subclinical hypothyroidism remains conflicting.^115,116^ A 2018 analysis from the TRUST trial, including 185 participants aged over 65, found that normalization of TSH with LT4 did not result in significant changes in carotid intima-media thickness (CIMT).^116,117^ These findings contrast with those of an earlier, smaller RCT of 45 participants with subclinical hypothyroidism, which reported a CIMT reduction of 0.09 mm (95% CI: .06–.11) after six months of achieving stable euthyroidism.^117^

## Current recommendations on the treatment of subclinical hypothyroidism

*Table 4* summarizes the recommendations of major international societies regarding the management of subclinical hypothyroidism.^104,118,119^ While guidelines recommend considering hypothyroid symptoms when initiating treatment, a secondary analysis of the TRUST trial showed symptom improvement even among participants receiving placebo.^120^ Overall, although some differences exist among societies, the major guidelines are broadly aligned. The divergence in international guidance likely reflects the limited strength of the available evidence. Notably, all major recommendations rely on relatively weak data, as no large RCT has been sufficiently powered to determine whether treatment provides clear cardiovascular benefits.^109^ While guidelines strive to be evidence-based, societies may differ in how they evaluate the strength of evidence, and in some areas, recommendations are made based on expert opinions, which invariably differ across organizations. This further highlights the current unmet need for adequately powered RCTs to determine whether treatment of subclinical hypothyroidism confers cardiovascular benefit. However, given that such trials have not been conducted to date and may not be feasible in the future, emulation trials or large prospective cohorts may be a pragmatic alternative to inform clinical practice. Moreover, while guidelines increasingly emphasize a personalized approach to managing subclinical hypothyroidism, such as using age-specific TSH reference ranges and consideration of comorbidities, future guidelines may also incorporate data from omics-based and genetic approaches as advances in these fields continue to emerge.

**Table 4:** Management of Subclinical Hypothyroidism^a^: Summary of ETA, NICE, and ATA Guidelines

TSH levels	European Thyroid Association^118^	National Institute for Health and Care Excellence^119^	American Thyroid Association^104^
**TSH < 10 mIU/L**	Age ≤70 years: if presence of hypothyroid symptoms, initiate LT4. If absence of hypothyroid symptoms, observe and repeat TFT in 6 months • Age >70 years: observe and repeat TFT in 6 months	Age <65 years: Consider a 6-month trial of LT4 if TSH above reference range but <10 mIU/L on two separate occasions 3 months apart and presence of hypothyroid symptoms	Consider LT4 if presence of hypothyroid symptoms, positive TPOAb, or evidence of atherosclerotic cardiovascular disease, heart failure, or associated risk factors for these diseases
**TSH ≥ 10 mIU/L**	Age ≤70 years: initiate LT4 Age >70 years: Consider LT4 if presence of clear hypothyroid symptoms or high vascular risk	Consider LT4 if TSH ≥ 10 mIU/L on two separate occasions 3 months apart	Consider LT4

ETA, European Thyroid Association, NICE, National Institute for Health and Care Excellence, ATA, American Thyroid Association, TSH, thyroid stimulating hormone, LT4, levothyroxine treatment, TFT, thyroid function tests.

^a^Subclinical hypothyroidism refers to individuals with elevated TSH and serum thyroxine within the reference range.

## Conclusions

Thyroid dysfunction, in particular overt hypothyroidism, can be regarded as a modifiable risk factor for CVD, the leading cause of death worldwide. However, the lack of adequately powered RCTs assessing whether hormone replacement therapy can improve cardiovascular outcomes in SCTD has rendered it challenging to develop clear evidence-based guidelines. The use of omics-based research and genetic studies holds future potential for clinical translation by enabling personalized care, tailoring treatment, and identifying which individuals with thyroid disorders may benefit most from intervention. Moreover, the conduct of larger clinical trials and the use of emulation methods may help resolve uncertainties and guide clinical practice in the future. A better understanding of the relationship between thyroid dysfunction and cardiovascular risk factors may contribute to more effective strategies for addressing the CVD epidemic.

## Supplementary Material

ehag248_Supplementary_Data

## Declarations

### Data Availability

No data were generated or analysed for or in support of this paper.
