# Supplementary material for: Thyroid dysfunction and cardiovascular disease
Source: Eur Heart J. 2026 Apr 17;47(21):2606–23. doi: 10.1093/eurheartj/ehag248 (PMC13225879; doi:10.1093/eurheartj/ehag248)
Supplement: ehag248_Supplementary_Data [file ehag248_supplementary_data.docx]

**Supplementary Materials**

**Title: Thyroid dysfunction and cardiovascular disease**

Heba Alwan^1,2^*, Ola Hysaj^1,3^*, Baris Gencer^1,4^, Leonidas Duntas^,5^, Nicolas Rodondi^1,6^

*Contributed equally

**Thyroid Status: a biochemical definition**

The thyroid is an endocrine gland responsible for the production and secretion of thyroid hormones.^1^ Approximately 80% of the hormones secreted by the thyroid gland is thyroxine (T4), which is largely inactive, while about 10% is triiodothyronine (T3), the biologically active form.^2^ Secretion of thyroid hormones is regulated by a feedback system known as the hypothalamic-pituitary-thyroid (HPT) axis.^3^

Thyroid function tests include measuring concentration levels of TSH, free T4 (FT4) and T3 that are routinely assessed in clinical practice using highly sensitive third-generation immunoassays.^4^ Current assays offer a detection threshold of TSH as low as 0.01 mU/L allowing a clear-cut discrimination of the normal thyroid function (euthyroid state) and thyroid dysfunction.^4^ The reference ranges of thyroid function tests provide the basis for the diagnosis and treatment of thyroid disease. However, no studies were conducted to establish clinical thresholds at which the risk of adverse consequences increases, and at when treatment is effective in reducing that risk. The current reference ranges are based on the 95% confidence interval from a healthy young population. Individuals with TSH, FT4, or T3 concentration values that fall within the lowest or highest 2.5% of this distribution are considered outside the normal range and are classified as abnormal.^4^

Over the past years, there has been a debate about whether thyroid test reference ranges should be reevaluated based on adverse clinical outcomes instead of being determined solely through statistical analysis.^5-7^ Despite emerging evidence reporting that the clinical consequences of abnormal thyroid function may extend even within the current reference ranges of TSH and FT4, no consensus has been reached, and current reference ranges continue to guide clinical practice.^6-8^

Thyroid dysfunction represents a continuum from overt disease to subclinical dysfunction. Overt hyperthyroidism is characterized by decreased serum TSH and increased FT4.^9^ Subclinical hyperthyroidism (Shyper) refers to abnormally low TSH concentrations with FT4 and total or free T3 concentrations within the reference range.^9,10^ Conversely, overt hypothyroidism is characterized by elevated TSH and decreased FT4 reflecting insufficient thyroid hormone production. Shypo refers to abnormally increased serum TSH concentrations with FT4 and total or free T3 concentrations within the reference range.^11^

Population-based studies report that the prevalence of overt hypothyroidism ranges from 0.2% to 5.3%, whereas Shypo affects approximately 4% to 17.5% of adults, with the highest prevalence among older adults.^12-15^ The prevalence of overt and subclinical hyperthyroidism ranges from 0.7 to 1.8% and from 0.6 to 15%, respectively.^13,16,17^This variability may be explained by the different characteristics of the studied populations (e.g., different iodine status) and the different assays of thyroid function used and reference ranges applied.

**Supplementary Table 1. Key Sources of Heterogeneity in Studies Examining Thyroid Dysfunction and Cardiovascular Outcomes**

| Source of Heterogeneity | Description | Potential Impact on Study Results |
| --- | --- | --- |
| Age distribution | Older adults have higher baseline TSH | Attenuated associations, especially for CHD and HF; weaker effect sizes in older cohorts. |
| Iodine sufficiency | Population differences in iodine intake influence thyroid autoimmunity and TSH distribution. | Stronger associations in iodine-deficient regions; weaker associations in iodine-replete populations. |
| Assay variability | Earlier studies used less sensitive TSH/FT4 assays (first generation); variation persists across laboratories. | Non-differential misclassification → bias toward the null; inconsistent classification of subclinical states. |
| Diagnostic thresholds | TSH and FT4 cutoffs for subclinical dysfunction vary between cohorts and guidelines. | Inconsistent classification of subclinical states → inconsistent effect estimates across studies. |
| Population comorbidity burden | Differences in cardiovascular risk factors, medication use, and history of CVD | Confounding that may obscure thyroid-related effects, especially in older or multimorbid groups. |
| Follow-up duration | Variation in length and frequency of follow-up assessments. | Shorter follow-up may underestimate long-term CVD risk related to thyroid dysfunction. |
| Outcome definitions | Differences in definitions of CVD endpoints: AF detection, CHD criteria, or HF adjudication. | Inconsistent outcome ascertainment → heterogeneity in effect sizes. |
| Treatment of thyroid dysfunction | Overt thyroid dysfunction is more prone to have been treated compared to subclinical thyroid dysfunction | Not excluding thyroid treatment users or accounting for it may underestimate CVD risk particularly in overt thyroid dysfunction |

CHD: coronary heart disease; HF: heart failure; TSH: thyroid stimulating hormone; FT4: free thyroxine; CVD: cardiovascular disease; AF: atrial fibrillation

**References**

1. Cooper David S, Ladenson PW. *The Thyroid Gland. In Greenspan's Basic and Clinical Endocrinology*. 10th edition. ed; 2018.

2. van der Spek AH, Fliers E, Boelen A. The classic pathways of thyroid hormone metabolism. Molecular and Cellular Endocrinology 2017;**458**:29-38.

3. Chiamolera MI, Wondisford FE. Minireview: Thyrotropin-releasing hormone and the thyroid hormone feedback mechanism. Endocrinology 2009;**150**(3):1091-1096.

4. Baloch Z, Carayon P, Conte-Devolx B, Demers LM, Feldt-Rasmussen U, Henry JF, LiVosli VA, Niccoli-Sire P, John R, Ruf J, Smyth PP, Spencer CA, Stockigt JR. Laboratory medicine practice guidelines. Laboratory support for the diagnosis and monitoring of thyroid disease. Thyroid 2003;**13**(1):3-126.

5. Biondi B. The normal TSH reference range: what has changed in the last decade? J Clin Endocrinol Metab 2013;**98**(9):3584-7.

6. Chaker L, Baumgartner C, den Elzen WP, Collet TH, Ikram MA, Blum MR, Dehghan A, Drechsler C, Luben RN, Portegies ML, Iervasi G, Medici M, Stott DJ, Dullaart RP, Ford I, Bremner A, Newman AB, Wanner C, Sgarbi JA, Dörr M, Longstreth WT, Jr., Psaty BM, Ferrucci L, Maciel RM, Westendorp RG, Jukema JW, Ceresini G, Imaizumi M, Hofman A, Bakker SJ, Franklyn JA, Khaw KT, Bauer DC, Walsh JP, Razvi S, Gussekloo J, Völzke H, Franco OH, Cappola AR, Rodondi N, Peeters RP. Thyroid Function Within the Reference Range and the Risk of Stroke: An Individual Participant Data Analysis. J Clin Endocrinol Metab 2016;**101**(11):4270-4282.

7. Xu Y, Derakhshan A, Hysaj O, Wildisen L, Ittermann T, Pingitore A, Abolhassani N, Medici M, Kiemeney L, Riksen NP, Dullaart RPF, Trompet S, Dörr M, Brown SJ, Schmidt B, Führer-Sakel D, Vanderpump MPJ, Muendlein A, Drexel H, Fink HA, Ikram MK, Kavousi M, Rhee CM, Bensenor IM, Azizi F, Hankey GJ, Iacoviello M, Imaizumi M, Ceresini G, Ferrucci L, Sgarbi JA, Bauer DC, Wareham N, Boelaert K, Bakker SJL, Jukema JW, Vaes B, Iervasi G, Yeap BB, Westendorp RGJ, Korevaar TIM, Völzke H, Razvi S, Gussekloo J, Walsh JP, Cappola AR, Rodondi N, Peeters RP, Chaker L. The optimal healthy ranges of thyroid function defined by the risk of cardiovascular disease and mortality: systematic review and individual participant data meta-analysis. Lancet Diabetes Endocrinol 2023;**11**(10):743-754.

8. Cappola AR, Arnold AM, Wulczyn K, Carlson M, Robbins J, Psaty BM. Thyroid function in the euthyroid range and adverse outcomes in older adults. J Clin Endocrinol Metab 2015;**100**(3):1088-96.

9. Biondi B, Kahaly GJ. Cardiovascular involvement in patients with different causes of hyperthyroidism. Nat Rev Endocrinol 2010;**6**(8):431-43.

10. Paschou SA, Bletsa E, Stampouloglou PK, Tsigkou V, Valatsou A, Stefanaki K, Kazakou P, Spartalis M, Spartalis E, Oikonomou E, Siasos G. Thyroid disorders and cardiovascular manifestations: an update. Endocrine 2022;**75**(3):672-683.

11. Nylen E, Zaloga G, Becker K, Burman K, Wartofsky L, Muller B. Endocrine therapeutics in critical illness. Principles and Practice of Endocrinology and Metabolism, 3rd edition. Philadelphia: Lippincott Williams & Wilkins 2001:2108-21.

12. Åsvold BO, Vatten LJ, Bjøro T. Changes in the prevalence of hypothyroidism: the HUNT Study in Norway. European Journal of Endocrinology 2013;**169**(5):613-620.

13. Garmendia Madariaga A, Santos Palacios S, Guillén-Grima F, Galofré JC. The incidence and prevalence of thyroid dysfunction in Europe: a meta-analysis. J Clin Endocrinol Metab 2014;**99**(3):923-31.

14. Li J, Li Y, Shi X, Teng D, Teng X, Teng W, Shan Z. Prevalence and risk factors of hypothyroidism after universal salt iodisation: a large cross-sectional study from 31 provinces of China. BMJ Open 2023;**13**(2):e064613.

15. Taylor PN, Albrecht D, Scholz A, Gutierrez-Buey G, Lazarus JH, Dayan CM, Okosieme OE. Global epidemiology of hyperthyroidism and hypothyroidism. Nat Rev Endocrinol 2018;**14**(5):301-316.

16. Ursem SR, Boelen A, Bruinstroop E, Elders PJM, Gussekloo J, Poortvliet RKE, Heijboer AC, den Elzen WPJ. A systematic review of subclinical hyperthyroidism guidelines: a remarkable range of recommendations. European Thyroid Journal 2024;**13**(3):e240036.

17. Zhang X, Wang X, Hu H, Qu H, Xu Y, Li Q. Prevalence and Trends of Thyroid Disease Among Adults, 1999-2018. Endocrine Practice 2023;**29**(11):875-880.
